# Considering an Approach for Assessing the Relevance of Tattoo-associated Health Risk from an Overall Toxicological Perspective

**Published:** 2018-05

**Authors:** Yongbum KWON, Do Gyun LEE

**Affiliations:** Dept. of Environmental Engineering, Incheon National University, Incheon, South Korea

## Dear Editor-in-Chief

Tattooing is performed by injecting coloring agents (e.g., black, white, blue, green, red and yellow) into the skin using needles. Especially among youngsters, the practice of skin tattoos, including cosmetic tattoos or permanent makeup, has become increasingly popular in recent years and been common worldwide. However, the students aged between 14–22 yr old were less knowledgeable about the potential health risk of tattooing, for instance, infectious disease, skin allergies and other medical complications due to the lack of emphasis on school education (1). More than 70% of tattooed individuals suffered from tattoo-associated skin problems or systemic reactions such as burning, itching, erythema, and eczema immediately after tattooing (2). Coloring agents in tattoo inks (commonly a mixture of small organic pigments, water, and isopropyl alcohol) contain metals, nanoparticles (NPs), and polycyclic aromatic hydrocarbons (PAHs), which may pose a risk to human health (3).

Metals in tattoo inks are used to create colors with various hues, brightness, and vividness; for instance, cobalt (Co), cadmium (Cd) and mercury (Hg) are the main components of colors for green, blue and red, respectively. Chromium (Cr), nickel (Ni) and Co were the metals detected in tattoo inks at the highest concentrations with the range of Cr: 315-147229, Ni: 37.5-9592, and Co: 2.78–6439 ng/g, respectively, though the concentrations recommended should not be more than 5 ppm of each metal in consumer products or 1 ppm for health protection (4). In addition, considerable amounts of NPs (at least one dimension <100 nm) were found in tattoo inks (5). Carbon black NPs in a black ink (the most frequent tattoo color) is considered as a potential carcinogen (group 2B) due to their high reactivity (5) and black inks are possibly transported into the human body through lymphatic system (6). Titanium dioxide (TiO_2_) NPs in white pigment was genotoxic in vivo (7). Tattoo inks contain a wide variety of PAHs such as benzo(a)pyrene (group 1; carcinogenic to humans), benzo(a)anthracene, benzo(k)fluoranthene, benzo(b)fluoranthene, chrysene, and naphthalene (8). In the commercial black tattoo inks, PAHs were present at concentrations of 0.14–201 μg/g (3). Remarkably, benzo(a)pyrene, one of the most potent PAH carcinogens, was detected at the highest concentration of 1.02 μg/g among the analyzed tattoo ink samples, which is 200 times higher than the level by the Council of Europe (0.005 μg/g).

However, there are no recommendations for benzo(a)anthracene, benzo(k)fluoranthene, benzo(b)fluoranthene, chrysene and naphthalene, classified as Group 2B (8).

“Are existing guidelines for the safety of tattoo inks enough for evaluating the toxicological effects of tattoo inks on human health?” In fact, tattoo inks can contain simultaneously metal, NPs and PAHs (3, 9). Especially in a black ink, tattoo inks commonly include both NPs and PAHs due to the production by controlled combustion (2, 8). To date, there has been little research studied on assessing overall toxicity of tattoo inks as a mixture of substances including toxic metals, NPs, PAHs, and other unknown substances. Production and transformation of byproducts and chemical reaction in the mixture form of tattoo inks in human body could contribute toward the antagonistic or the synergistic toxicity. As some fraction of toxicologically active substances in tattoo inks are also unknown, the assessment of overall toxicity in tattoo inks to human is used to determine the potential for overall toxicological impact on human health. Thus, the development of an approach for comprehensively assessing the overall toxicological impact of tattoo-associated health risk in tattoo inks should be further considered. Meanwhile, a strict and detailed legislation for tattoo inks manufacturing, tattooing practice, and systemic toxicity evaluation of tattoo ink ingredients should be required for public health and safety. Simultaneously, health education about tattooing in school will help to increase students’ awareness of tattoo-associated health risk.
